# Morin-functionalized pHEMA cryogel membranes for combating MDR *Escherichia coli* and MRSA: antibacterial efficacy and molecular docking study

**DOI:** 10.1007/s00210-026-04976-9

**Published:** 2026-01-11

**Authors:** Özge Öztürk Cimentepe, Mehmet Cimentepe, Kemal Dogan, Metin Yildirim

**Affiliations:** 1 Department of Pharmacology, Faculty of Pharmacy, Harran University, Sanliurfa, Türkiye; 2 Department of Pharmaceutical Microbiology, Faculty of Pharmacy, Harran University, Sanliurfa, Türkiye; 3 Advanced Technology Research and Application Center, Harran University, Sanliurfa, Türkiye; 4 Department of Biochemistry, Faculty of Pharmacy, Harran University, Sanliurfa, Türkiye

**Keywords:** Morin, PHEMA, Cryogel membrane, MRSA, MDR *Escherichia coli*

## Abstract

In this study, morin-loaded poly(2-hydroxyethyl methacrylate) (pHEMA) cryogels were successfully synthesized and characterized. The swelling behavior of the cryogels was evaluated, and their biocompatibility was assessed against L929 fibroblast cells. The antibacterial efficacy of the cryogel membranes against multidrug-resistant (MDR) *Escherichia coli (E. coli) and methicillin-resistant* *Staphylococcus aureus* (MRSA) was investigated using disk diffusion and time–kill assays, while bacteria-induced morphological alterations were visualized by SEM. Molecular docking studies were performed to elucidate the interaction mechanisms of morin with 4DUH, 4DX5, 4WUB, and 5NC5 proteins, yielding docking scores of − 5.578, − 4.142, − 6.955, and − 4.607 kcal/mol, respectively. The strongest binding affinity was observed for 4WUB, supported by the lowest docking score and a Glide emodel value of − 58.834, indicating a stable ligand–protein complex. The synthesized cryogels exhibited a high swelling ratio of 97.89 ± 14.21% and demonstrated excellent biocompatibility, with L929 cell viability ranging from 86 to 100% after 48 h of exposure, even at the highest tested dose of 1.5 mg, confirming the absence of cytotoxic effects. The MM1 and MM2 cryogel membranes showed pronounced antibacterial activity against MDR *E. coli,* producing inhibition zones of 16.1 mm and 17.6 mm, respectively. In time–kill assays, MM1 exhibited inhibition rates of 48.3% against MRSA and 91.6% against MDR *E. coli* at the 8th hour, while MM2 achieved enhanced inhibition rates of 57.1% and 99.6%, respectively. Overall, these findings indicate that morin-loaded pHEMA cryogel membranes represent promising antibacterial platforms for the treatment of infected wounds and for medical device surface coatings to prevent bacterial colonization and infection.

## Introduction

Antibiotic resistance is a global public health problem primarily driven by the misuse and overuse of antibiotics, and its threat continues to escalate worldwide. Data published by the World Health Organization (WHO) indicate that antimicrobial resistance (AMR) is associated with approximately 1.27 million deaths annually worldwide (Sivarajan et al. 2025). *Escherichia coli (E. coli)*, commonly recognized as a gut commensal bacterium, is frequently encountered in clinical settings and has developed resistance to several antibiotic classes, including β-lactams, aminoglycosides, and (fluoro)quinolones (Erdoğan and Akbulut 2021). Drug resistance not only increases the economic burden of treatment, but also leads to severe clinical outcomes, including increased mortality rates. Forecasts suggest that antimicrobial resistance could become responsible for up to 10 million deaths each year by 2050 in the absence of effective mitigation strategies (Aswal et al. 2025). AMR is a multifactorial and complex phenomenon, arising from diverse bacterial mechanisms. Recent studies emphasize that targeting bacterial signaling pathways and resistance mechanisms may represent a promising strategy to combat drug resistance (Elshobary et al. 2025; Abebe and Birhanu 2023; Moo et al. 2020). The discovery and development of new antibiotics is a time-consuming and costly process, requiring extensive research and development efforts, which significantly limits rapid therapeutic advancement (Miethke et al. 2021; Livermore, et al. 2011; Mantravadi et al. 2019; Atanasov et al. 2021). Therefore, the development of novel and effective antimicrobial approaches has become critically important.

Medicinal plants have been extensively used in the treatment of various diseases and have long been incorporated into traditional and folkloric medicine (Amangeldinova et al. 2025; Srinivasan et al. 2001). In recent years, increasing attention has been directed toward phytochemicals derived from medicinal plants due to their broad pharmacological potential. Among these compounds, flavonoids, flavonols, and saponins have been widely investigated for the treatment of numerous diseases (Rajput et al. 2021). Morin, a member of the flavanol subclass, is naturally present in several plant families, including Rosaceae and Fagaceae, with particularly high abundance in the Moraceae family (Rajput et al. 2021; Jain et al. 2025). Morin is known to exhibit a wide range of therapeutic properties, including antioxidant (Vijay and Perumal 2025), antibacterial (Sales et al. 2025), anti-inflammatory (Arjsri et al. 2025), and anticancer (Yıldırım et al. 2025a) activities.

From a physicochemical perspective, morin exhibits poor water solubility, appears as a yellow-colored compound, and is readily soluble in alcohol (Vijay and Perumal 2025; Yazdanshenas and Gharib 2017). Its low aqueous solubility significantly limits its therapeutic bioavailability and clinical applicability. To overcome these limitations, nanotechnology-based delivery systems have been widely explored. In this context, various carriers such as polymeric nanoparticles (Shree et al. 2023), lipid-based nanoparticles (Bagade et al. 2023), hydrogels (Micale et al. 2020; Mirrezaei et al. 2020), and cryogels (Yildirim et al. 2025a) have been employed to improve the delivery and efficacy of poorly soluble phytochemicals.

Poly(2-hydroxyethyl methacrylate), p(HEMA), is a polymeric material synthesized from HEMA monomers and is well known for its excellent biocompatibility, which has led to its widespread use in biomedical applications, particularly in contact lenses (Farazin and Gheisizadeh 2025; Nečas et al. 2025). Cryogels, synthesized under sub-zero temperature conditions, offer a unique advantage as delivery platforms for temperature-sensitive phytochemicals and drugs, as their fabrication process minimizes thermal degradation (Patole et al. 2025; Okoye et al. 2024).

Unlike previous studies focusing on morin in its free form or loaded into conventional polymeric systems, the present work introduces a macroporous, three-dimensional cryogel membrane platform, enabling both localized antibacterial activity and potential applicability as a bioactive scaffold. This integrated strategy provides a novel approach at the intersection of antibacterial biomaterials and tissue-regenerative platforms. Therefore, p(HEMA)-based cryogels represent a promising platform for the effective delivery of bioactive compounds such as morin. To the best of our knowledge, this is the first study in which morin has been incorporated into pHEMA cryogel membranes, and their antibacterial activity against methicillin-resistant *Staphylococcus aureus* (MRSA) and multi drug resistance (MDR) *E. coli* has been systematically evaluated. In the present work, pHEMA cryogel membranes incorporating morin were successfully fabricated and structurally characterized by Fourier transform infrared spectroscopy (FTIR) and scanning electron microscopy (SEM). The physicochemical behavior of the membranes was further evaluated through swelling measurements. Their antibacterial performance was examined against MRSA and MDR *E. coli* using disk diffusion and time-kill kinetic assays. Following the identification of the most sensitive bacterial strain, molecular docking analyses were performed to elucidate the possible interactions between morin and essential bacterial target proteins involved in cellular processes.

## Material and methods

### Preparation of morin-loaded and unloaded cryogel membranes

Morin-loaded and unloaded cryogels (M) were prepared according to a previously reported synthesis protocol (Yildirim et al. 2025b). Briefly, 2.475 mL of HEMA monomer and 0.8 mL of 1-vinylimidazole were mixed in a tube until a homogeneous solution was obtained. In a separate tube, 100 mg of MBAAm was dissolved in distilled water, after which the two solutions were combined. Polymerization was initiated by the addition of APS and TEMED, and the reaction mixture was incubated overnight at − 20 °C to allow cryogel formation. Following synthesis, the obtained membranes were washed with an ethanol–water mixture to remove unreacted components. After drying, the membranes were loaded with morin at concentrations of 200 (MM1) and 400 µg/mL (MM2).

### Characterization

#### FTIR analysis

The functional groups of the synthesized M, MM1, and MM2 membranes were analyzed using FTIR spectroscopy (IRTracer-100, Shimadzu Corporation).

#### SEM analysis

The surface morphologies of M, MM1, and MM2 membranes were examined using SEM (SEM, ZEISS EVO50). Prior to SEM analysis, the samples were lyophilized and subsequently gold-coated using a sputter coater (Luxor, BE).

#### Swelling degree

The swelling behavior of the membranes was evaluated gravimetrically. Briefly, dried membranes were weighed to obtain the dry weight and then immersed in distilled water in Petri dishes for 24 h at room temperature. After incubation, excess surface water was gently removed, and the swollen weight was recorded.

### Biocompatibility assessment

To evaluate the effects of the synthesized gels, both morin-loaded and unloaded, on cell viability in relation to their potential biomedical applications, a cell viability assay was performed. L929 fibroblast cells (ATCC) were cultured at 37 °C in a humidified incubator with 5% CO₂. Cells were seeded at a density of 2 × 10^5^ cells per well and allowed to attach for 24 h. The prepared gels were then applied at concentrations between 0.5 and 1.5 mg and incubated with the cells for 48 h. Cell viability was evaluated using the MTT assay by adding MTT solution and incubating for 4 h, followed by dissolution of the resulting formazan crystals in 100 µL of dimethyl sulfoxide (DMSO). Absorbance was measured using a microplate reader to determine cell viability (Yildirim et al. 2025c).

### In vitro morin release study

In vitro release studies of morin from the cryogel membranes were carried out in phosphate buffer at 37 °C. Briefly, 100 mg of morin-loaded cryogel membrane was immersed in 10 mL of phosphate buffer solution. At predetermined time intervals, aliquots were withdrawn and the amount of released morin was quantified by UV–Vis spectrophotometry at 222 nm using a Shimadzu UV-1280 spectrophotometer. The loading efficiency of morin was calculated according to previously reported methods (Zagni et al. 2023).

### Evaluation of antibacterial activity

The antibacterial activity of M, MM1, and MM2 cryogel membranes was evaluated using agar diffusion and plate counting assays in accordance with previously reported methods (Hudzicki 2009; Li et al. 2023). The analyses were conducted against MRSA (ATCC 43300) and MDR *E. coli* (BAA 3337) strains.

For the agar diffusion assay, bacterial suspensions adjusted to 0.5 McFarland standard (1.5 × 10^8^ CFU/mL) were spread onto Mueller–Hinton agar plates, followed by placement of sterilized cryogels. Vancomycin and gentamicin served as reference antibiotics, and inhibition zones were measured after 18–24 h of incubation.

To specifically evaluate the antibacterial activity of the synthesized gels in a liquid medium, a time-kill assay was performed. Antibacterial efficacy was further quantified using the plate counting method. Bacterial cultures (1 × 10^6^ CFU/mL) prepared in tryptic soy broth were incubated with sterilized cryogels, while untreated wells served as controls. After 8 h of incubation, samples were serially diluted, plated on Mueller–Hinton agar, and viable colonies were enumerated to determine bacterial survival (Yildirim et al. 2024, 2025d; Zulfiqar et al. 2024).

#### Morphological changes of bacteria

After completion of the plate counting assay, the M, MM1, and MM2 cryogel membranes were aseptically transferred into 24-well plates. Bacteria adhered to the cryogel surfaces were fixed using 3% glutaraldehyde. The samples were subsequently dehydrated through a graded ethanol series (50%- 100%). Finally, bacterial morphology and surface-associated structural changes were analyzed by SEM (Yildirim et al. 2025a).

### Molecular docking

Molecular docking studies were performed using the Maestro 13.8 software package. The crystallographic structures of the 24 kDa and N-terminal domains of *Escherichia coli* DNA gyrase subunit B (PDB IDs: 4DUH and 4WUB), along with the multidrug efflux transporter AcrB (PDB IDs: 4DX5 and 5NC5), were retrieved from the Protein Data Bank. Ligand preparation was conducted employing the OPLS-4 force field, and docking simulations were executed using the Glide extra-precision (XP) mode. The resulting docking scores were reported in kcal/mol, with increasingly negative values corresponding to stronger predicted ligand–protein interactions (Yıldırım et al. 2025b).

### Statistical analysis

All experiments were performed in triplicate, and the results are presented as mean ± standard deviation (SD). Statistical analyses were conducted using GraphPad Prism 10 (GraphPad Software, San Diego, CA, USA). Differences among multiple groups were evaluated by one-way analysis of variance (ANOVA), followed by Tukey’s multiple comparison post hoc test. A p value of less than 0.05 was considered statistically significant. Levels of statistical significance are indicated as **p < 0.01 and ****p < 0.0001.

## Results and discussion

### Characterization

**Fig. 1:**
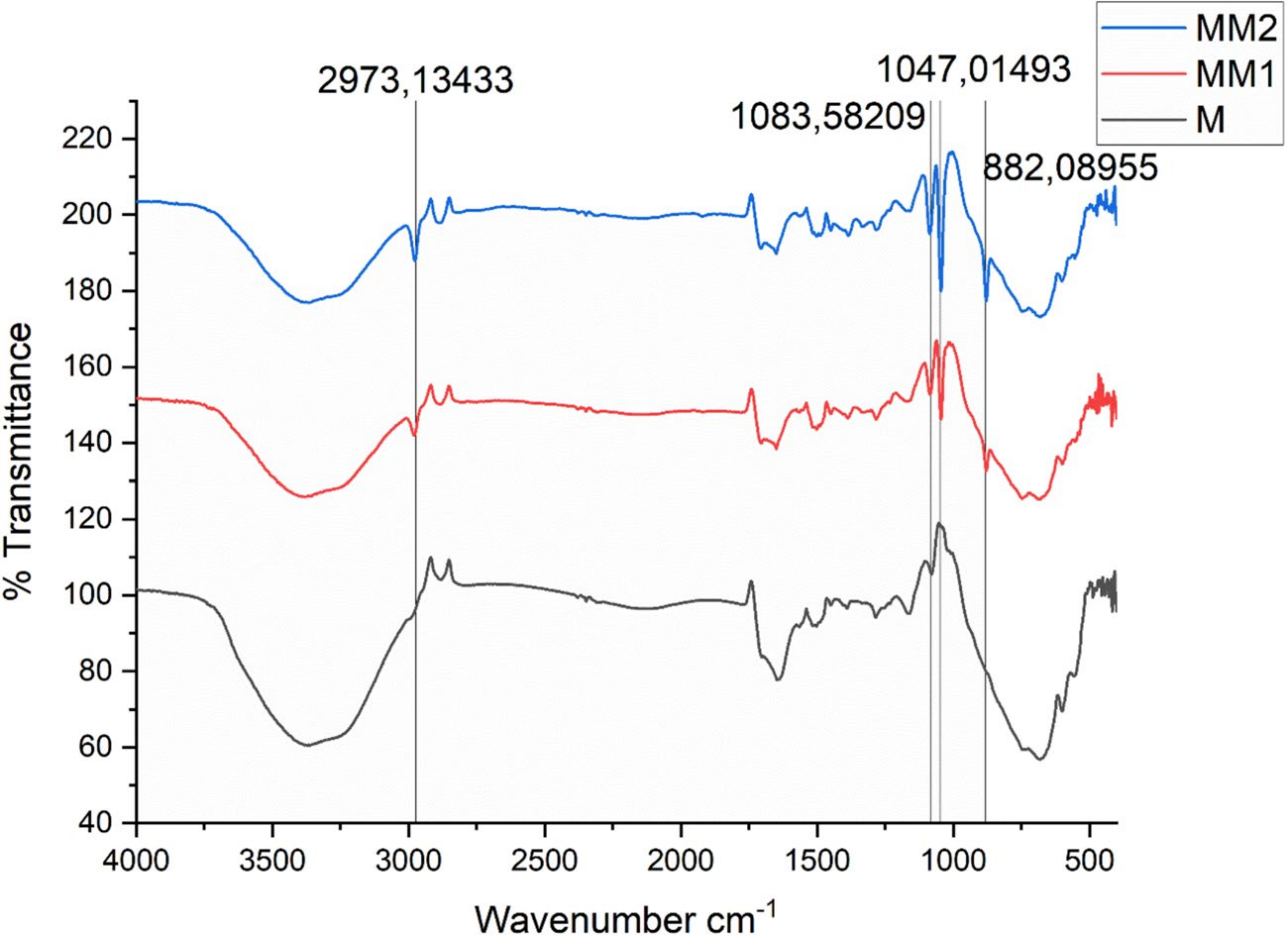
FTIR spectra of pristine and morin-loaded cryogel membranes. FTIR spectra of M, MM1 and MM2 membranes are shown in the range of 4000–500 cm⁻^1^

The FTIR spectra of M, MM1, and MM2 cryogel membranes were recorded to confirm the chemical structure of the cryogels and the successful incorporation of morin into the polymer matrix (Fig. 1).

In all spectra, the broad absorption band observed in the range of 3200–3500 cm⁻^1^ is attributed to the O–H stretching vibrations, originating from the hydroxyl groups of p(HEMA) and morin. The intensity of this band increased noticeably in MM1 and MM2, indicating enhanced hydrogen bonding interactions due to morin incorporation.

The characteristic peak observed at approximately 2973 cm⁻^1^ corresponds to the C–H stretching vibrations of aliphatic –CH₂ and –CH₃ groups, confirming the polymer backbone of the p(HEMA)-based cryogels. This peak is present in all samples, demonstrating that the polymer framework remained intact after morin loading.

The absorption band detected around 1083 cm⁻^1^ is assigned to C–O–C stretching vibrations, characteristic of the ester groups in the p(HEMA) structure. In MM1 and MM2 cryogels, slight shifts and intensity variations of this band were observed, suggesting intermolecular interactions between morin and the cryogel matrix.

A distinct peak at approximately 1047 cm⁻^1^ is attributed to C–O stretching vibrations, which are more pronounced in the morin-loaded cryogels, further confirming the presence of morin within the cryogel membranes.

Additionally, the band observed near 882 cm⁻^1^ is associated with out-of-plane bending vibrations of aromatic C–H groups, which can be attributed to the aromatic ring structures of morin. The increased intensity of this peak in MM1 and MM2, compared to the unloaded M cryogel, provides further evidence of successful morin incorporation.

Overall, the FTIR results confirm that morin was successfully incorporated into the p(HEMA) cryogel membranes without altering the fundamental polymeric structure. The observed peak shifts and intensity changes indicate hydrogen bonding and physical interactions, rather than chemical bond formation, suggesting that morin is physically entrapped within the cryogel network.

#### Swelling behavior of the membranes

**Fig. 2:**
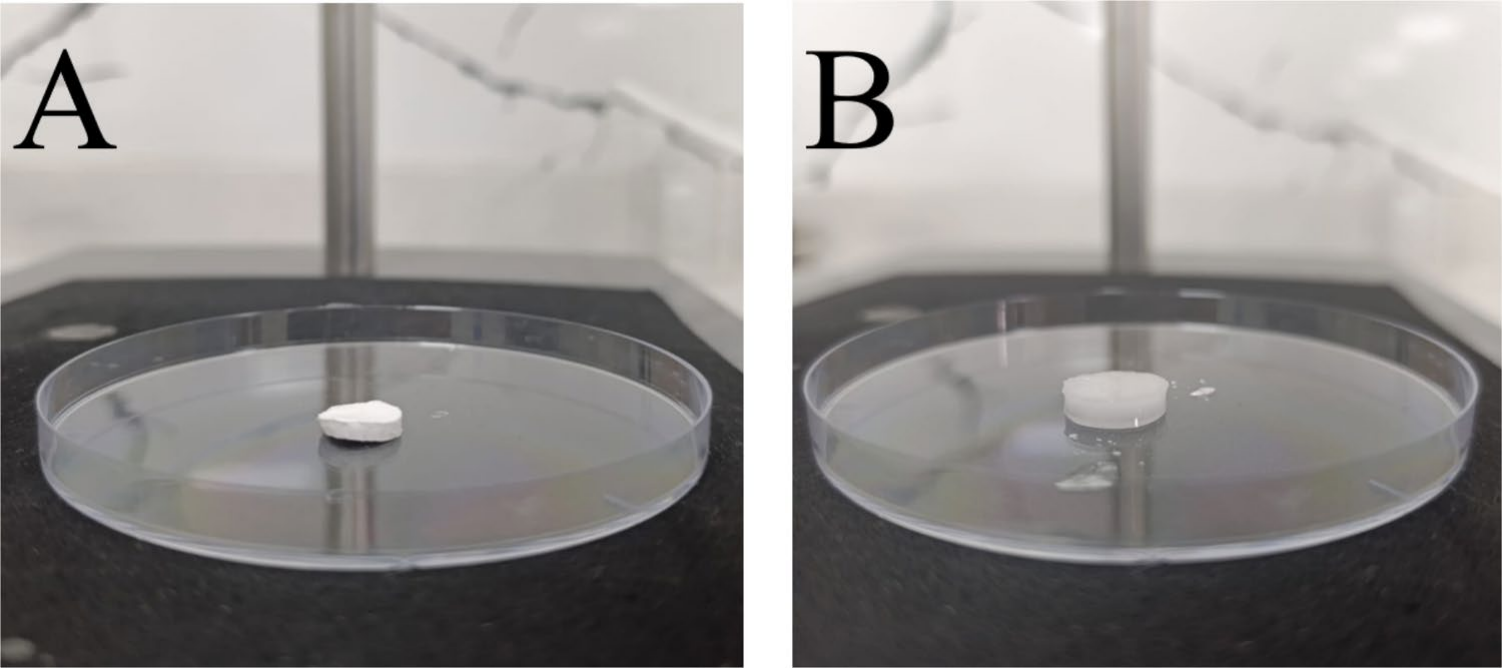
Visual representation of the swelling test: (**A**) dry state of the sample and (**B**) wet state after 24 h of incubation in the swelling medium. A noticeable increase in the sample’s volume and thickness is observed in the wet state

As a result of the swelling experiments, the dry and swollen weights of the membranes were calculated as 0.1072 ± 0.0042 g and 0.2126 ± 0.0227 g, respectively. The swelling degree was calculated individually for each sample, yielding an average swelling degree of 97.89 ± 14.21% (mean ± SD, *n* = 3) (Fig. 2).

### Biocompatibility assessment

Evaluating the effects of the synthesized cryogel membranes on fibroblast cells is of critical importance for their potential wound-healing applications following morin loading. Although pHEMA-based membranes and hydrogels are generally recognized as biocompatible, cytotoxicity assessment must be performed for each synthesis batch due to the possible presence of residual monomers or synthesis-related variations.

**Fig. 3:**
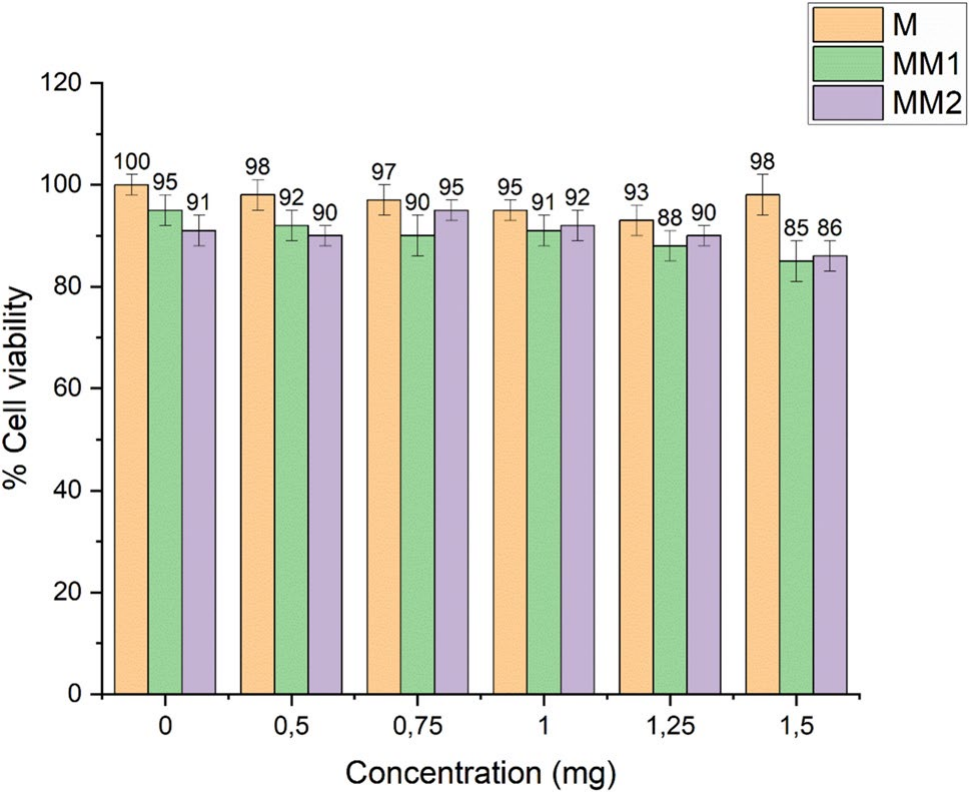
Cell viability of L929 fibroblast cells treated with cryogel membranes. L929 fibroblast cells were exposed to unloaded membranes (M) and morin-loaded membranes (MM1 and MM2) at different concentrations (0–1.5 mg) for 48 h. All tested membrane formulations maintained high cell viability, indicating good biocompatibility, even at the highest applied dose. Data are expressed as mean ± SD (*n* = 3)

In this study, both morin-loaded and unloaded cryogel membranes were applied to L929 fibroblast cells for 48 h, and cell viability was evaluated using the MTT assay. The results demonstrated that cell viability ranged between 86 and 100% across all tested conditions. The highest applied dose (1.5 mg) resulted in a cell viability of 86%, indicating no significant cytotoxic effect (Fig. 3).

Based on these findings, the synthesized cryogel membranes were confirmed to be biocompatible, supporting their suitability for potential wound-healing applications.

### In vitro release studies and loading efficiency 

**Fig. 4:**
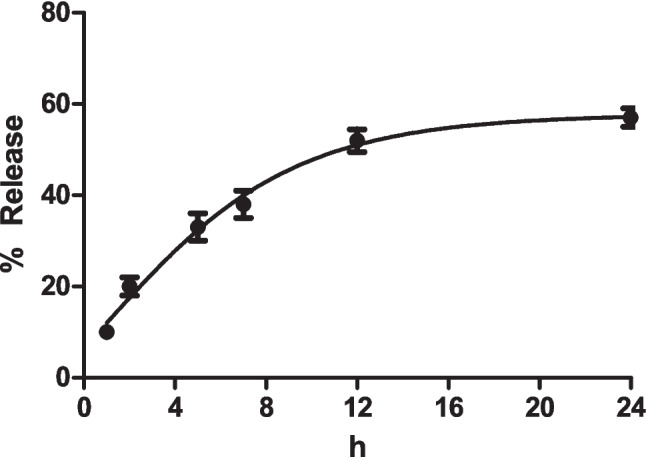
Release profiles of morin from the cryogel membranes. Data are presented as mean ± SD

An in vitro release study was conducted to evaluate the release behavior of morin from the prepared cryogel membrane. During the 24 h release period, approximately 33% of morin was released within the first 5 h, followed by 52% at 12 h, reaching 57% at 24 h, indicating a sustained and controlled release profile rather than an initial burst release (Fig. 4). Furthermore, the loading efficiency of morin within the synthesized cryogel membranes was determined to be 78.5%. This relatively high loading efficiency can be attributed to favorable interactions between the polyphenolic structure of morin and the functional groups present in the cryogel matrix, such as hydrogen bonding and physical entrapment within the interconnected porous structure. These findings are consistent with previously reported values for flavonoid-loaded hydrogel and cryogel-based delivery systems and support the observed time-dependent antibacterial activity.

### In vitro antibacterial activity

**Fig. 5:**
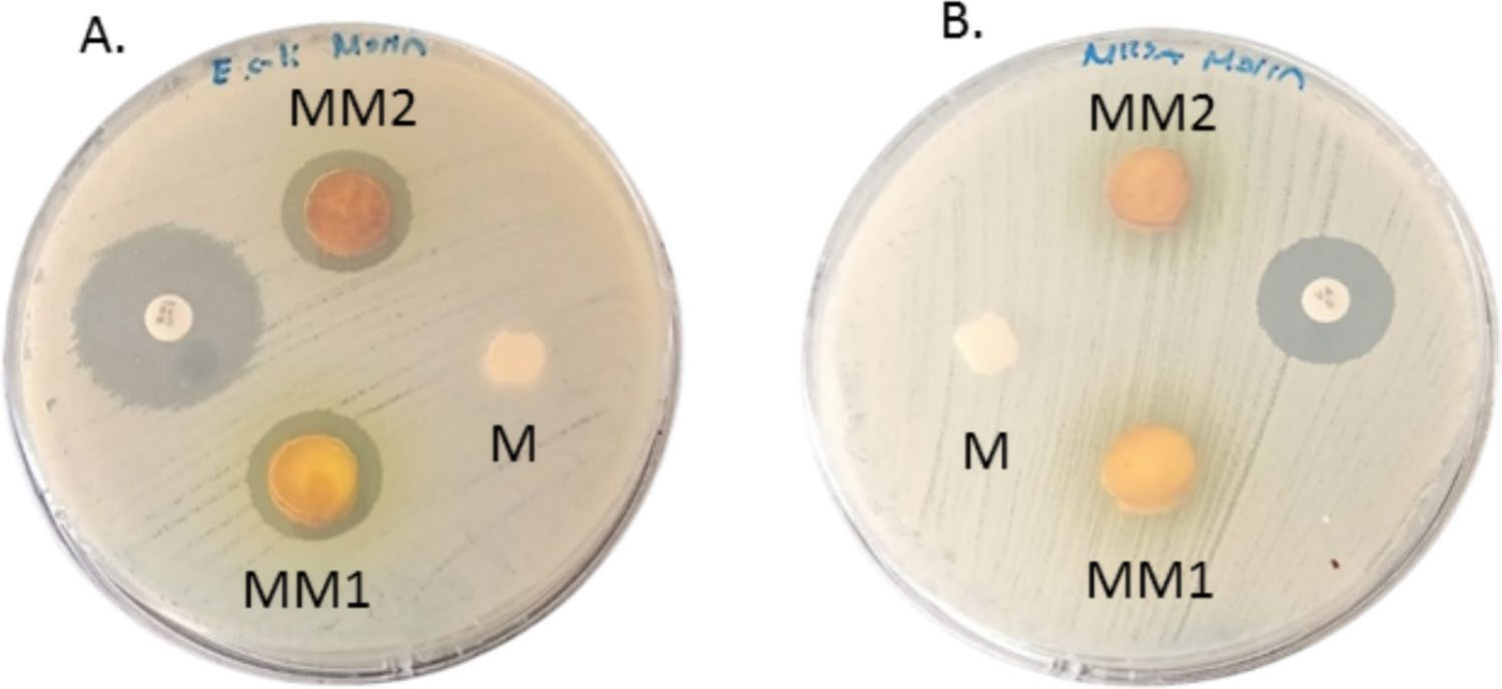
Disk diffusion assay showing the inhibitory effects of M, MM1, and MM2 cryogel membranes against *MDR E. coli* (**A**) and MRSA (**B**)

The inhibition zones formed by MM1, MM2, and M cryogels against MRSA and MDR *E. coli* strains are presented in Fig. 5. The M cryogel did not exhibit antibacterial activity against either MRSA or MDR *E. coli*. Similarly, morin-loaded MM1 and MM2 cryogels, at different morin concentrations, did not produce inhibition zones against the MRSA strain.

In contrast, both MM1 and MM2 cryogels demonstrated pronounced antibacterial activity against MDR *E. coli*, forming inhibition zones with diameters of 16.1 mm and 17.6 mm, respectively. These findings indicate that morin incorporation significantly enhanced the antibacterial efficacy of the cryogels, particularly against MDR *E. coli*.

**Fig. 6:**
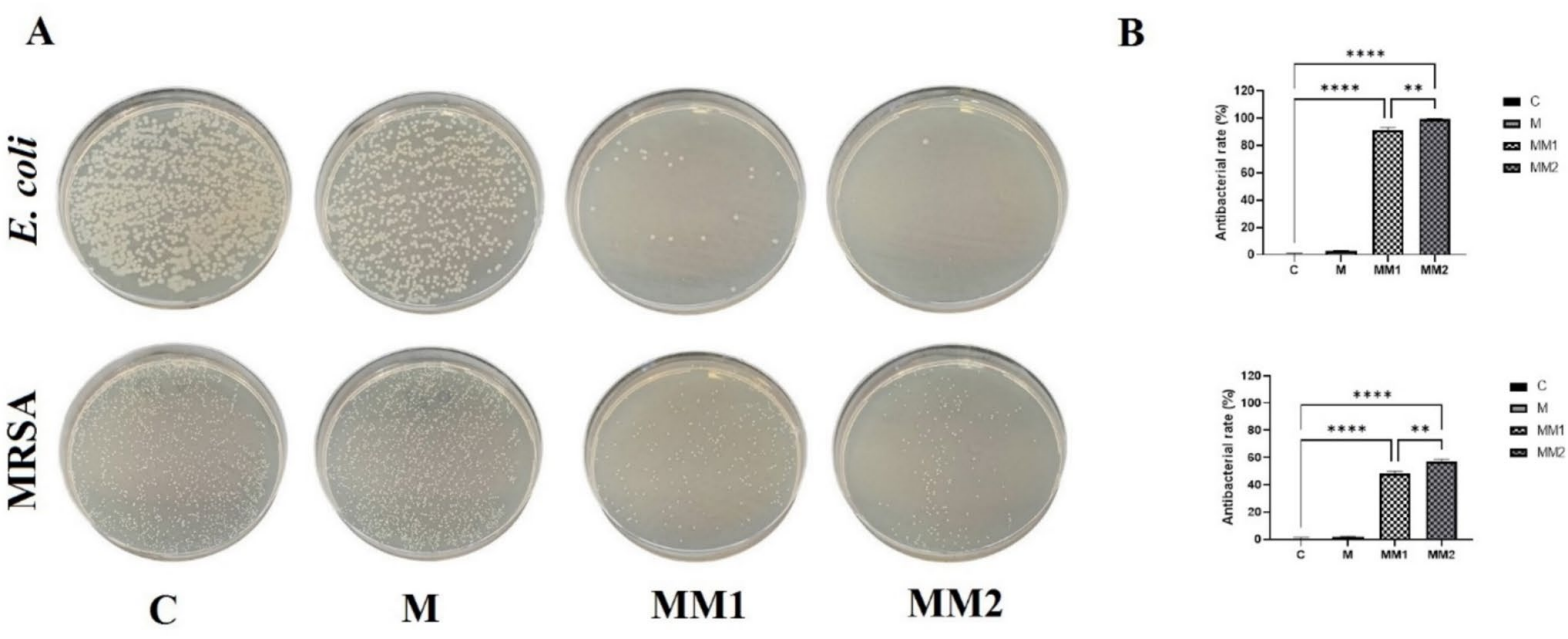
Antibacterial performance of cryogel membranes. (**A**) Colony formation of *MDR E. coli* and MRSA. (**B**) Corresponding antibacterial inhibition rates under different experimental conditions

The antibacterial performance of cryogel membranes against MRSA and *MDR E. coli* is crucial for effective infected wound management. Accordingly, the antibacterial efficacy of various cryogel formulations was assessed against MRSA and *MDR E. coli* using the plate counting assay. The M cryogel membranes showed no detectable antibacterial effect against either bacterial strain. In contrast, the MM1 formulation exhibited notable inhibitory activity, with bacterial reduction rates of 48.3% for MRSA and 91.6% for *MDR E. coli* after 8 h of incubation. Likewise, the MM2 cryogel membranes demonstrated superior antibacterial efficacy, achieving inhibition rates of 57.1% against MRSA and 99.6% against *MDR E. coli* under identical experimental conditions (Fig. 6).

The partial inhibition observed against MRSA in suspension culture may be plausibly associated with the swelling behavior of the cryogel membrane matrix in the aqueous phase, which can promote pore expansion and thereby influence the release kinetics of the incorporated active compound. This swelling-induced structural change may facilitate a gradual and sustained release over time, leading to enhanced antibacterial effects under liquid-phase conditions rather than in solid media (Çetin and Denizli 2015). Following 8 h of incubation with MRSA and MDR *E. coli*, bacterial morphological changes were further evaluated using SEM. In the M cryogel group, both MRSA and MDR *E. coli* cells exhibited smooth, spherical, and intact cell morphologies. In contrast, bacteria exposed to MM1 and MM2 cryogels showed pronounced morphological alterations, including roughened surfaces and disrupted bacterial membranes, likely resulting from electrostatic interactions between the positively charged cryogel surfaces and negatively charged bacterial cell walls, ultimately leading to bacterial cell death.

Chronic wounds infected with MDR bacteria represent a serious public health challenge, significantly contributing to increased morbidity and mortality. The rapid emergence of MDR pathogens has further exacerbated this issue, rendering conventional antibiotic therapies largely ineffective. Consequently, there is an urgent need to develop innovative, antibiotic-free therapeutic strategies that do not solely rely on conventional antimicrobial agents (Shah et al. 2025; Wang et al. 2020). Due to their simple synthesis procedures and favorable physicochemical properties, p(HEMA)-based cryogel wound dressings have been widely applied in various biomedical applications (Usal et al. 2019). Moreover, the incorporation of bioactive agents during synthetic polymer fabrication can impart antibacterial properties to cryogel systems.

In the present study, based on the fundamental characteristics of MDR bacteria–infected wounds, we aimed to develop a therapeutic cryogel membrane loaded with the natural flavonoid morin as an antibacterial agent. Previous studies have reported similar strategies; for instance, Şahiner et al. demonstrated antimicrobial activity using p(HEMA) and poly(tannic acid) nanoparticle-containing supermacroporous cryogels (Sahiner et al. 2017). Consistent with our results, Diken Gür et al*.* observed strong antibacterial effects of lysozyme-loaded p(HEMA-MAH) cryogel membranes against *E. coli* strains (Diken Gür et al. 2021). Moreover, our earlier investigations demonstrated that cryogel systems incorporating natural flavonoids exhibit antibacterial activity against *S. aureus*, MRSA, *E. coli*, and MDR *E. coli* strains (Yildirim et al. 2024, 2025e).

In the present work, disk diffusion assays revealed that morin-loaded cryogels exhibited antibacterial activity against MDR *E. coli*, whereas no inhibition zone was detected against MRSA. However, plate counting results at the 8th hour demonstrated that morin-loaded cryogel membranes exerted strain-dependent inhibitory effects against both MRSA and MDR *E. coli*. The antibacterial efficacy of morin is attributed to its aromatic ring structures and abundant hydroxyl groups, which facilitate bacterial aggregation, induce oxidative stress, and promote strong interactions with bacterial cell membranes, ultimately resulting in cell death (Kováč et al. 2022). Additionally, these structural features enhance the penetration capability of morin into dense and resistant bacterial biofilms (Bouchelaghem et al. 2022).

Overall, MM1 and MM2 cryogels exhibited significantly stronger antibacterial activity against MDR *E. coli* compared to MRSA, with the antibacterial effect increasing in a concentration-dependent manner (Sales et al. 2025). These findings suggest that morin-loaded cryogel membranes represent a promising next-generation antibacterial wound dressing for the treatment of MDR *E. coli–infected wounds*, highlighting the clinical potential of morin as a natural antimicrobial agent.

**Fig. 7:**
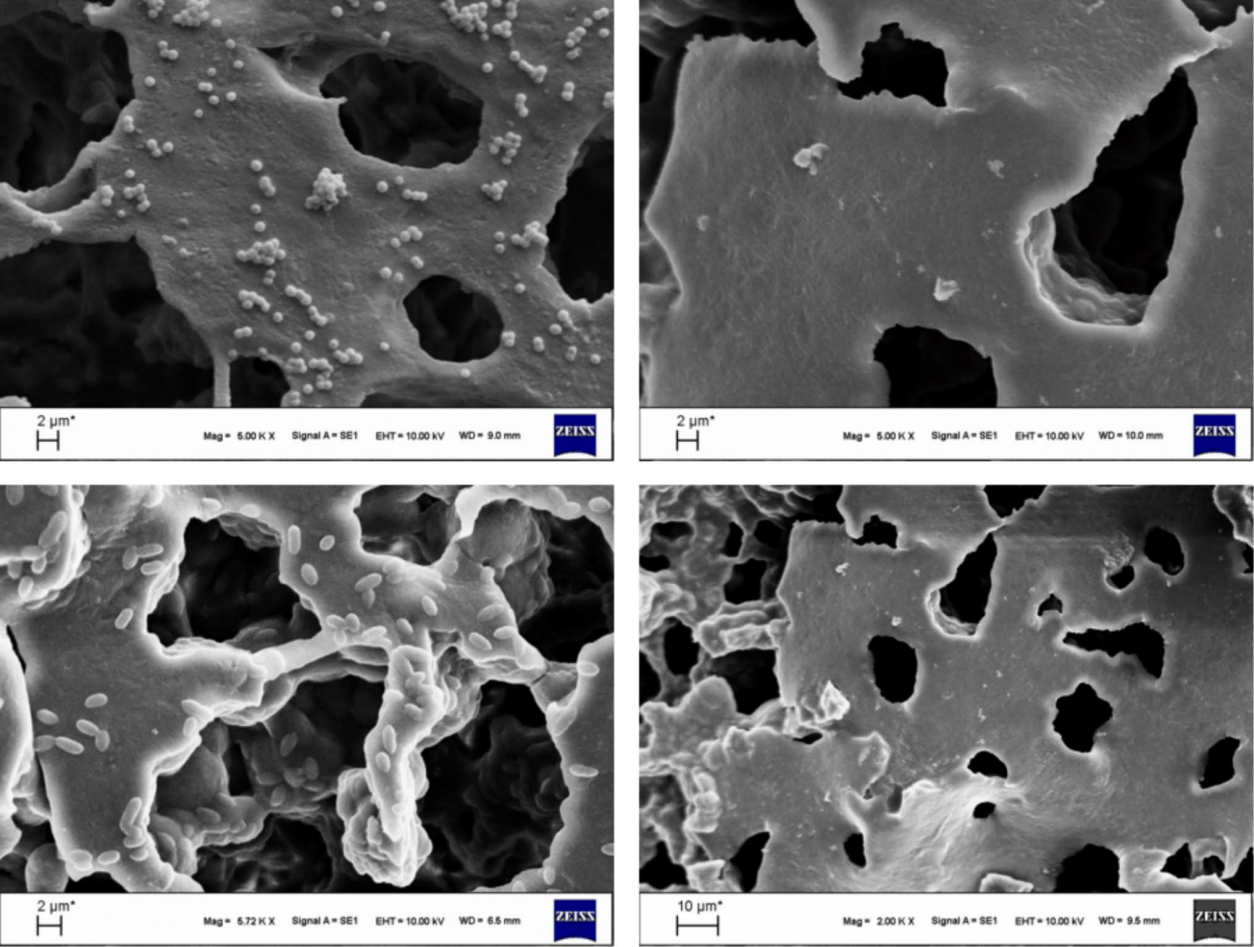
SEM micrographs showing bacterial adhesion on unloaded and morin-loaded cryogel membranes. SEM images illustrate the interaction of cryogel membranes with MRSA and MDR *E. coli*. The upper left panel shows MRSA cells adhered to the M, while the upper right panel represents MRSA exposed to the MM2. The lower left panel displays MDR *E. coli* on the M, whereas the lower right panel shows MDR *E. coli* on the morin-loaded membrane MM2. Compared to the unloaded membranes, the morin-loaded cryogels exhibit a marked reduction in bacterial adhesion along with evident morphological deformation and surface damage, indicating the antibacterial effectiveness of morin incorporation. Scale bars: 2 µm and 10 µm

The cryogels exhibited a highly interconnected macroporous architecture, a defining characteristic of cryogel-based systems that facilitates bacterial adhesion in the absence of antibacterial agents. In the morin-unloaded membranes, bacteria readily attached to and colonized the cryogel surfaces. MRSA cells maintained a typical spherical morphology with smooth and intact cell walls, while MDR *E. coli* displayed its characteristic rod-shaped, flattened, and structurally preserved morphology, indicating normal cellular integrity and metabolic activity (Fig. 7).

In contrast, the morin-loaded cryogel membranes induced pronounced morphological and structural damage in both bacterial strains. These effects can be mechanistically attributed to the synergistic action of the cryogel matrix and morin. The swelling behavior of the cryogels in aqueous environments leads to pore expansion, enabling the controlled and sustained release of morin into the surrounding medium. Once released, morin—rich in aromatic rings and multiple hydroxyl groups—is able to establish strong interactions with bacterial cell membranes, including hydrogen bonding, hydrophobic interactions, and π–π stacking with membrane-associated components.

### Molecular docking

**Fig. 8:**
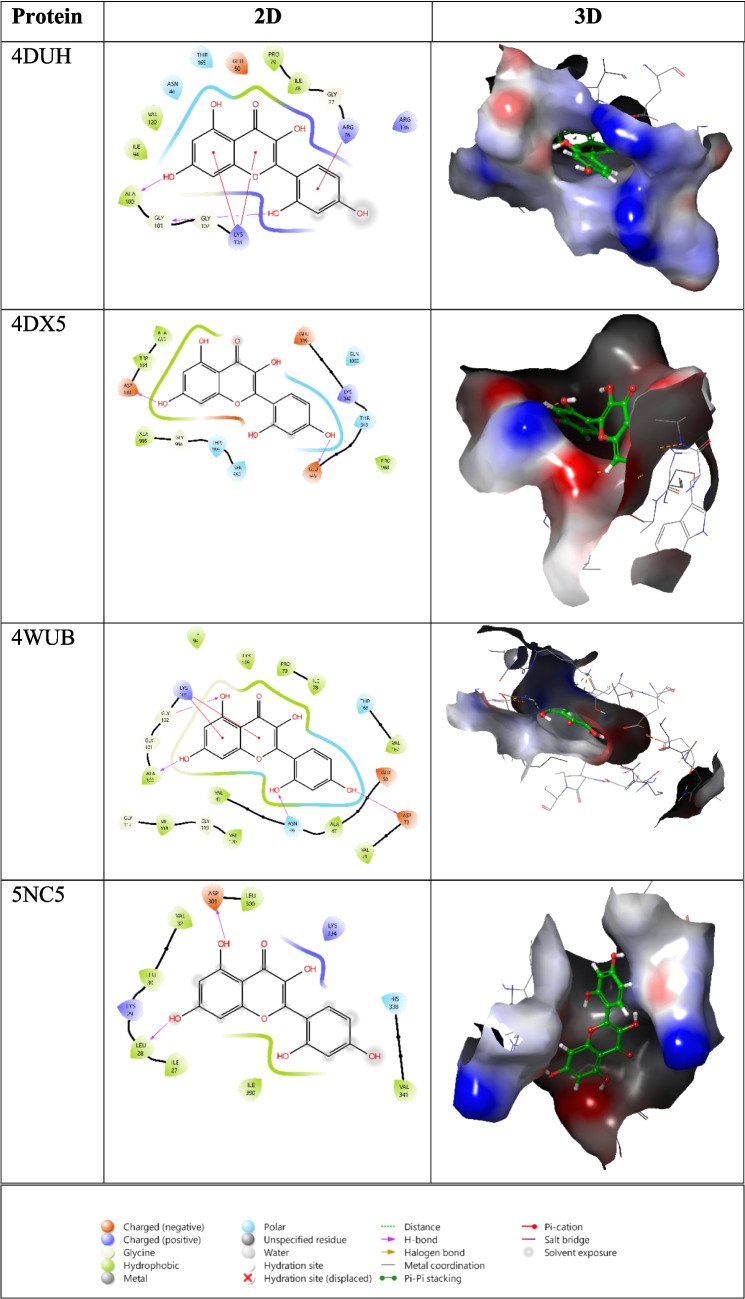
Visualization of the 2D and 3D binding modes of morin in the active sites of the selected bacterial target proteins

The interactions of morin with *E. coli* DNA gyrase subunit B (PDB IDs: 4DUH and 4WUB) as well as the multidrug efflux pump AcrB (PDB IDs: 4DX5 and 5NC5) were investigated using molecular docking analysis. The docking scores ranged from − 6.955 to − 4.142 kcal/mol (Table 1). Morin exhibited the strongest binding affinity toward 4WUB with a docking score of − 6.955 kcal/mol, while the weakest interaction was observed with a docking score of − 4.142 kcal/mol. Analysis of the 4WUB–morin complex revealed that the hydroxyl (–OH) groups of morin formed hydrogen bonds with ALA100, ASP73, and ASN46. In addition, a π–cation interaction was observed between the ligand and LYS103. For the 4DUH–morin complex, π–cation interactions with ARG76 and LYS103 were detected, along with a hydrogen bond interaction with ALA100. In the case of the 5NC5–morin complex, hydrogen bonds were formed with ASP301 and LEU28, whereas in the 4DX5–morin complex, hydrogen bonding interactions were observed with ASP633 and GLU346 (Fig. 8). It should be emphasized that the molecular docking results presented in this study provide supportive and preliminary insights into possible interactions between morin and the selected bacterial targets. The obtained docking scores (− 6.955 to − 4.142 kcal/mol) indicate moderate binding affinity and should not be interpreted as definitive evidence of a specific antibacterial mechanism. Molecular docking is a predictive approach that does not account for protein flexibility, solvent effects, or intracellular conditions. Therefore, these results are intended to complement the experimental antibacterial findings rather than to establish a mechanistically conclusive mode of action.

**Table 1:** Molecular docking scores and glide emodel values of morin

Protein	Docking score	Glide emodel
4DUH	−5.578	−48.754
4DX5	−4.142	−41.139
4WUB	−6.955	−58.834
5NC5	−4.607	−43.275

## Conclusion

In this study, morin was successfully incorporated into cryogel membranes and structurally confirmed by FTIR analysis. The synthesized cryogel membranes exhibited excellent biocompatibility, as demonstrated by the L929 fibroblast cell viability ranging from 86 to 100% following 48 h of exposure, even at the highest tested dose of 1.5 mg, confirming the absence of cytotoxic effects. These findings indicate that the morin-loaded cryogels are safe for potential biomedical and wound-related applications.

The cryogel membranes also displayed a high swelling capacity, with an average swelling degree of 97.89 ± 14.21% (*n* = 3), reflecting their strong water absorption ability. The high swelling property favors wound dressing use by enabling effective exudate absorption and maintaining a moist environment conducive to wound healing and antimicrobial action.

Antibacterial studies revealed that morin-loaded cryogels exhibited concentration-dependent antibacterial activity against MDR *E. coli*, producing clear inhibition zones in disk diffusion assays, whereas morin-unloaded cryogels showed no antibacterial effect. Although no measurable inhibition zones were observed against MRSA in disk diffusion tests, time-kill assays demonstrated effective, concentration-dependent bacterial eradication of both MDR *E. coli* and MRSA, emphasizing the superior antibacterial performance of the cryogel system under moist conditions that better mimic the wound microenvironment.

SEM analyses further supported these findings by revealing pronounced bacterial membrane disruption and severe morphological damage after treatment with morin-loaded cryogels. In addition, molecular docking studies provided mechanistic insight into the antibacterial activity of morin, showing favorable binding affinities ranging from − 6.955 to − 4.142 kcal/mol toward key bacterial targets. The strongest interaction was observed with *E. coli* DNA gyrase subunit B (PDB ID: 4WUB, − 6.955 kcal/mol), supporting the experimental antibacterial results at the molecular level.

Overall, the combined outcomes of high biocompatibility (≥ 86% cell viability), substantial swelling capacity (~ 98%), strong antibacterial efficacy, and favorable molecular docking interactions indicate that morin-loaded cryogel membranes represent a promising multifunctional antibacterial platform. Taken together, the findings highlight this system as a highly promising platform for next-generation wound dressings aimed at controlling infections caused by multidrug-resistant bacteria. While the present study demonstrates promising in vitro antibacterial and biocompatibility results, it is limited by the absence of in vivo validation and long-term release evaluation under physiological conditions. Future studies focusing on infected wound models, extended release kinetics, and optimization of morin loading levels will be essential to further advance the therapeutic potential of morin-loaded cryogel membranes.

## Data Availability

All source data for this work (or generated in this study) are available upon reasonable request.
